# Supplementary material for: Clinical Trials of Immune Checkpoint Inhibitors in Hepatocellular Carcinoma
Source: J Clin Med. 2021 Jun 16;10(12):2662. doi: 10.3390/jcm10122662 (PMC8234948; doi:10.3390/jcm10122662)
Supplement: Supplementary file 1 [file jcm-10-02662-s001.zip › jcm-1244067-supplementary.pdf]

Supplementary Search String S1

Search String S1:

(Hepatocellular OR liver) AND (carcinoma OR cancer) AND checkpoint inhibitor*; (Hepatocellular OR liver) AND (carcinoma OR cancer) AND checkpoint blockade; (Hepatocellular OR liver) AND (carcinoma OR cancer) AND Tremelimumab; (Hepatocellular OR liver) AND (carcinoma OR cancer) AND Ipilimumab; (Hepatocellular OR liver) AND (carcinoma OR cancer) AND (Nivolumab OR MDX-1106 OR BMS-936558); (Hepatocellular OR liver) AND (carcinoma OR cancer) AND (Pembrolizumab OR Lambrolizumab OR MK-3475); (Hepatocellular OR liver) AND (carcinoma OR cancer) AND (Tislelizumab OR BGB-A317); (Hepatocellular OR liver) AND (carcinoma OR cancer) AND (Camrelizumab OR SHR-1210); (Hepatocellular OR liver) AND (carcinoma OR cancer) AND (Cemiplimab or REGN2810); (Hepatocellular OR liver) AND (carcinoma OR cancer) AND (Spartalizumab OR PDR001); (Hepatocellular OR liver) AND (carcinoma OR cancer) AND (Sintilimab or IBI308); (Hepatocellular OR liver) AND (carcinoma OR cancer) AND (Toripalimab OR JS001); (Hepatocellular OR liver) AND (carcinoma OR cancer) AND AMP-224; (Hepatocellular OR liver) AND (carcinoma OR cancer) AND (Atezolizumab or MSB0010718C); (Hepatocellular OR liver) AND (carcinoma OR cancer) AND (Durvalumab OR MEDI4736); (Hepatocellular OR liver) AND (carcinoma OR cancer) AND (Avelumab OR MSB0010718C); (Hepatocellular OR liver) AND (carcinoma OR cancer) AND (BMS935559 OR MDX-1105). All searches were restricted to publications in English. References lists of related review articles were manually checked for additional studies. In addition, we searched the ASCO and ESMO libraries for relevant conference abstracts.

Supplementary Table S1.

Ongoing studies with checkpoint inhibitors in advanced/metastatic hepatocellular carcinoma

Clinical trial.gov identifier	Treatment	Phase	Line of treatment	Patient characteristics	Number of patients	Primary end points	Estimated study completion
**PD-1/PD-L1 inhibitor, monotherapy**							
NCT03163992	**Pembrolizumab**	II	2. line	Not amenable to curative treatment or locoregional therapy or refractory to locoregional therapy<br>BCLC stage C or B<br>Child-Pugh A<br>Prior sorafenib	60	ORR	September 2020, recruiting
NCT03419481	**Pembrolizumab**	II	Refractory	Advanced, not amenable to curative surgery or loco-regional ablation<br>HBV+	30	RR	June 2021, recruiting
NCT03062358 (KEYNOTE-394)	**Pembrolizumab** + best supportive care<br>vs	III	2. line	Not amenable to curative treatment or locoregional therapy or	450	OS	June 2021, active, not recruiting

Clinical trial.gov identifier	Treatment	Phase	Line of treatment	Patient characteristics	Number of patients	Primary end points	Estimated study completion
	Placebo + best supportive care			refractory to locoregional therapy<br>BCLC stage B or C<br>Child-Pugh A<br>Prior sorafenib or oxaliplatin-based chemotherapy			
NCT03419897<br>(RATIONALE-208)	**Tislelizumab**	II	≥ 2. line	Not amenable for locoregional therapy<br>Child-Pugh A	228	ORR	September 2021, active, not recruiting,
NCT03412773<br>(RATIONALE-301)	**Tislelizumab**<br>vs<br>Sorafenib	III	1. line	BCLC C or B<br>Child-Pugh A	660	OS	May 2022, recruiting
NCT04564313	**Camrelizumab**	I	≥ 2. line	Recurrence after liver transplantation<br>Child Pugh A and B7<br>PS 0-2	20	ORR	July 2021, recruiting

Clinical trial.gov identifier	Treatment	Phase	Line of treatment	Patient characteristics	Number of patients	Primary end points	Estimated study completion
NCT03966209	**Toripalimab**	I	NA	Recurrence or metastases after transplantation not suitable for local treatment<br>Child-Pugh A<br>Prior sorafenib	20	SAE, acute graft rejection rate	April 2021, recruiting
NCT03949231	**Toripalimab**<br>vs<br>**Toripalimab** intrahepatic	III	1. line	Unresectable/metastatic not amenable for locoregional treatment<br>Child-Pugh A or B7	200	OS	January 2022, recruiting
**PD-1/PD-L1 inhibitor in combination with antiangiogenic drugs**							
NCT03382886 (NUANCE)	**Nivolumab** + bevacizumab	I	Any	Unresectable or metastatic<br>Child-Pugh A<br>Prior TKI	12	AE, MTD	March 2023, recruiting
NCT03418922	**Nivolumab** + lenvatinib	Ib	1. line	Unresectable, not eligible for resection or local ablation	30	DLT, safety<br>ORR	June 2020, active, not recruiting

**Clinical trial.gov identifier**	**Treatment**	**Phase**	**Line of treatment**	**Patient characteristics**	**Number of patients**	**Primary end points**	**Estimated study completion**
				Child-Pugh A			
NCT01658878 (CheckMate040)	**Nivolumab**<br>**Nivolumab** + **ipilimumab**<br>**Nivolumab** + cabozantinib<br>**Nivolumab** + **ipilimumab** + cabozantibnib<br>Sorafenib	I/II	Any	Not candidate for locoregional therapy<br>Child-Pugh A + B 7	620	Safety<br>ORR	Active not recruiting, partly published
NCT03841201	**Nivolumab** + lenvatinib	II	1. line	Unresectable, not eligible for resection or local ablation<br>Child-Pugh A	50	ORR, safety	July 2021, recruiting
NCT03439891	**Nivolumab** + sorafenib	II	1. line	Locally advanced/metastatic<br>Child-Pugh A or B7	40	MTD<br>ORR	May 2020, recruiting
NCT04393220	**Nivolumab**<br>+ bevacizumab	II, randomized	1. line	Inegible for local treatment	60	PFS, OS	April 2021, active, not recruiting

Clinical trial.gov identifier	Treatment	Phase	Line of treatmen t	Patient characteristics	Numbe r of patient s	Primary end points	Estimated study completio n
	Nivolumab<br>Bevacizumab			BCLC B or C<br>Child Pugh A			
NCT03347292	**Pembrolizumab** + regorafenib	I	1. line	BCLC stage C or B<br>Child-Pugh A	40	TEAE	June 2021, recruiting
NCT03211416	**Pembrolizumab** + sorafenib	I/II	1. line	Advanced or metastatic<br>Child-Pugh A<br>No prior sorafenib	27	ORR	September 2021, recruiting
NCT4442581	**Pembrolizumab** +<br>cabozantinib	II	1. line	Advanced<br>BCLC stage B or C<br>Child Pugh A	29	RR	September 2023, recruiting
NCT04696055	**Pembrolizumab** +<br>regorafenib	II	Any	Unresectable advanced<br>BCLC stage B or C<br>Child Pugh A<br>Prior PD1/PD-L1 inhibitor	119	ORR	November 2022, recruiting

**Clinical trial.gov identifier**	**Treatment**	**Phase**	**Line of treatmen t**	**Patient characteristics**	**Numbe r of patient s**	**Primary end points**	**Estimated study completio n**
NCT03713593 (LEAP -002)	Lenvatinib + **pembrolizumab**<br>vs<br>Lenvatinib + placebo	III	1. line	Not amenable for local therapy or refractory to local therapy<br>BCLC stage B or C<br>Child-Pugh A	750	PFS, OS	July 2022, recruiting
NCT04401800	**Tislelizumab** + lenvatinib	II	1. line	Advanced/ metastatic<br>BCLC B or C<br>Child Pugh A	66	ORR	September 2022, recruiting
NCT04443309	**Camrelizumab** + lenvatinib	I/II	1. line	not suitable for surgical or local therapy<br>BCLC B or C<br>Child Pugh A	53	ORR	August 2022, recruiting
NCT04014101	**Camrelizumab +** apatinib (VEGFR2 inhibitor)	II	1. line	Advanced<br>BCLC stage B or C3<br>Child-Pugh A<br>Prior sorafenib/rivastatin	40	ORR	October 2019, recruiting?

**Clinical trial.gov identifier**	**Treatment**	**Phase**	**Line of treatment**	**Patient characteristics**	**Number of patients**	**Primary end points**	**Estimated study completion**
NCT03463876	**Camrelizumab +** apatinib (VEGFR2 inhibitor)	II	1. or 2. line	Advanced<br>BCLC stage B or C<br>Child-Pugh A	190	ORR	October 2019, active, not recruiting
NCT03793725	**Camrelizumab +** apatinib (VEGFR2 inhibitor)	II	Any	Unresectable	30	R0 resection	January 2020, unknown
NCT04523662	**Camrelizumab** + apatinib + radiotherapy	II	Any	Not suitable for surgical resection or local treatment, patients with lymph or lung metastasis<br>Child Pugh A	27	PFS	August 2022, not yet recruiting
NCT03764293	**Camrelizumab +** apatinib (VEGFR2 inhibitor)<br>vs<br>Sorafenib	III	1. line	Not suitable for local therapy<br>BCLC stage B or C<br>Child-Pugh A	510	OS<br>PFS	December 2021, recruiting
NCT04618367	Oxaliplatin + 5 FU intra hepatic +	Not applicable	?<br>Refused sorafenib	No extrahepatic disease<br>Portal vein tumor thrombus	30	PFS	December 2021, recruiting

Clinical trial.gov identifier	Treatment	Phase	Line of treatment	Patient characteristics	Number of patients	Primary end points	Estimated study completion
	lenvatinib + **sintilimab**			Child Pugh A or B<br>PS 0-2			
NCT02988440	**Spartalizumab** + sorafenib	I	1. line	Advanced/metastatic not amenable for surgical or locoregional treatment<br>Child-Pugh A	20	AE	December 2019, completed
NCT04042805	**Sintilimab** + lenvatinib	II	1. line	BCLC B not amenable for resection or C<br>Child-Pugh score ≤7	566	ORR	August 2022, not yet recruiting
NCT04052152	**Sintilimab** + anlotinib (VEGFR/EGFR inhibitor)	II	1. line	BCLC B or C<br>Child-Pugh score ≤7	20	ORR<br>AE	December 2019, recruiting
NCT04411706	**Sintilimab** + apatinib + capecitabine	II	1.line	BCLC B and C<br>Child Pugh A	46	ORR	June 2021, recruiting
NCT04718909	**Sintilimab** + regorafenib<br>vs	II, randomized	2.line	Unresectable<br>Child Pugh A	180	PFS	June 2022, recruiting

Clinical trial.gov identifier	Treatment	Phase	Line of treatment	Patient characteristics	Number of patients	Primary end points	Estimated study completion
	Regorafenib						
NCT03794440	**Sintilimab** + IBI305 (anti-VEGF antibody)<br>Sorafenib	II/III	1. line	BCLC B or C<br>Child-Pugh score ≤7	595	OS, PFS	December 2022, active not recruiting
NCT04503902	**Toripalimab** + donafenib (multikinase inhibitor)	I/II	Any	Advanced/metastatic<br>Not suitable for surgery	46	DLT, ORR	December 2022, not yet recruiting
NCT04368078	**Toripalimab** + lenvatinib	II	2. line	Advanced<br>BCLC B or C<br>Child Pugh A	76	ORR	April 2022, recruiting
NCT04605796	**Toripalimab** + bevacizumab	II	1. line	BCLC B or C<br>Child Pugh A	60	TEAE, ORR	March 2021, recruiting
NCT04627363	Oxaliplatin + 5 FU intrahepatic + **toripalimab** + bevacizumab	II	1. line	BCLC B or C<br>Child Pugh A or B	30	PFS	June 2022, not yet recruiting

Clinical trial.gov identifier	Treatment	Phase	Line of treatment	Patient characteristics	Number of patients	Primary end points	Estimated study completion
NCT04523493	**Toripalimab** + lenvatinib<br>vs<br>Lenvatinib	III	1. line	Advanced<br>BCLC stage B or C<br>Child Pugh A + B7	486	OS, PFS	May 2024, recruiting
NCT04723004	**Toripalimab** + bevacizumab<br>vs<br>Sorafenib	III	1. line	Unsuitable surgery and local therapy<br>BCLC B or C<br>Child Pugh A	280	PFS, OS	August 2022, recruiting
NCT04732286	**Atezolizumab** + bevacizumab	III, b<br>single arm	1. line	Advanced/metastatic<br>Not amenable to surgery and local therapy	100	Discontinuation of atezolizumab due to AE	December 2022, not yet recruiting
NCT04487067 (AMETHISTA)	**Atezolizumab** + bevacizumab	III, b<br>single arm	1. line	Not amenable to surgery and local therapy<br>Child Pugh A	150	AE grade 3-5 bleeding	October 2022, recruiting
NCT03755791 (COSMIC-312)	**Atezolizumab** + cabozantinib<br>vs<br>Sorafenib	III	1. line	Not amenable to curative or locoregional therapy<br>BCLC B or C	740	PFS, OS	June 2021, recruiting

Clinical trial.gov identifier	Treatment	Phase	Line of treatment	Patient characteristics	Number of patients	Primary end points	Estimated study completion
	vs<br>Cabozantinib			Child-Pugh A			
NCT04770896 IMbrave251	**Atezolizumanab** + lenvatinib<br>vs<br>**Atezolizumab** + sorafenib<br>vs<br>Lenvatinib<br>vs<br>Sorafenib	III	Any	Advanced /metastatic<br>Unresectable<br>Child Pugh A<br>Prior atezolizumab + bevacizumab	554	OS	September 2024, not yet recruiting
NCT03970616	**Durvalumab** + tivozanib (VEGF inhibitor)	Ib/II	1. line	Metastatic<br>Child-Pugh A	42	TEAE	August 2021, recruiting
NCT04443322 (DULECT2020-1)	**Durvalumab** + lenvatinib	Single group, not applicable	1. line	Advanced/metastatic<br>Child Pugh A	20	PFS, RFS	December 2021, recruiting

Clinical trial.gov identifier	Treatment	Phase	Line of treatment	Patient characteristics	Number of patients	Primary end points	Estimated study completion
NCT03475953 (Regomune)	**Avelumab +** regorafenib	I/II<br>Including other tumor types	2. line	Advanced/metastatic<br>Child-Pugh A	362	RP2D<br>ORR	November 2021, recruiting
**PD-1/PD-L1 inhibitor in combination with CTLA-4 inhibitor**							
NCT04039607 (CheckMate 9DW)	**Nivolumab + ipilimumab** vs<br>Sorafenib/lenvatinib	III	1. line	Advanced HCC<br>Child-Pugh 5-6	1084	OS	September 2023, recruiting
NCT04401813	**Sintilimab** + IBI310 (CTLA-4 inhibitor)	I	Refractory	Advanced/metastatic	47	AE, ORR	April 2023, recruiting
NCT04720716	**Sintilimab** + IBI310 (CTLA-4 inhibitor)<br>vs<br>Sorafenib	III	1. line	BCLC B and C<br>Child Pugh A	490	OS, ORR	December 2023, recruiting

**Clinical trial.gov identifier**	**Treatment**	**Phase**	**Line of treatment**	**Patient characteristics**	**Number of patients**	**Primary end points**	**Estimated study completion**
NCT03482102	**Durvalumab + tremelimumab +** radiation	II<br>Including biliary tract cancer	2. line	Locally advanced/metastatic<br>Child-Pugh A + B7<br>Prior sorafenib	70	ORR	October 2021, recruiting
NCT02519348	**Durvaluma**b vs **Tremelimumab** vs **Durvalumab** + **Tremelimumab vs Durvalumab +** bevacizumab	II, randomized	2. line	Unresectable<br>Child-Pugh A-B7<br>Prior sorafenib	440	AE, DLT	December 2019, active, not recruiting
NCT03298451 (HIMALAYA)	**Durvalumab** vs **Durvalumab + tremelimumab** vs sorafenib	III	1. line	Not amenable to local therapy<br>Child-Pugh A	1310	OS	March 2020, recruiting
**PD-1/PD-L1 inhibitor in combination with other agents**							
NCT03059147	**Nivolumab** + SF1126 (Bromodomain inhibitor)	I	Any	Not candidate for local therapy<br>Child-Pugh A + B7<br>Prior sorafenib	14	DLT	October 2022, recruiting

Clinical trial.gov identifier	Treatment	Phase	Line of treatment	Patient characteristics	Number of patients	Primary end points	Estimated study completion
NCT03893695	**Nivolumab +**<br>GT90001 (ALK1 antibody)	I	≥ 2. line	BCLC B or C<br>Child-Pugh A	20	DLT	May 2020, active, not recruiting
NCT03655613	**Nivolumab** or CBT501 (PD-1 inhibitor) + CBT-101 (cMet inhibitor)	I/II	Any	Advanced/metastatic<br>Prior sorafenib	119	DLT	September 2020, unknown
NCT02423343	**Nivolumab** + galunisertib (Transforming growth factor-beta receptor I kinase inhibitor)	Ib/II	2. line	Recurrent/refractory<br>Elevated alpha-fetoprotein<br>Child-Pugh A<br>Prior sorafenib	75	MTD<br>PFS	Completed
NCT02859324	**Nivolumab** + CC-122 (E3 ligase inhibitor)	I/II	1–3. line	Unresectable<br>≤ 2 prior systemic therapies	50	DLT, AE<br>ORR	Completed
NCT02705105	**Nivolumab** + mogamulizumab (CC chemokine receptor 4 (CCR4) antibody)	I/II	≥ 2. line	Advanced or metastatic<br>Child-Pugh A	114	MTD<br>ORR	Completed

**Clinical trial.gov identifier**	**Treatment**	**Phase**	**Line of treatment**	**Patient characteristics**	**Number of patients**	**Primary end points**	**Estimated study completion**
NCT03695250	**Nivolumab + BMS-986205 (IOD1 inhibitor)**	I/II	≤ 2. line	Not amenable for curative treatment<br>Child-Pugh A	23		December 2021, recruiting
NCT03897543	**Nivolumab +**<br>ABX196 (Natural Killer T cell agonist)	I/II	≥2. line	Not amenable for curative treatment<br>Child-Pugh A	48	AE	January 2021, recruiting
NCT03071094	**Nivolumab +** Pexa-Vec (intratumoral oncolytic immunotherapy)	I/II	1. line	Advanced or metastatic<br>Child-Pugh A	30	DLT<br>ORR	October 2019, active not recruiting
NCT03781960	**Nivolumab** + abemaciclib	II	Any	Advanced/metastatic<br>Retinoblastoma expression positive	27	ORR	January 2021, suspended (due to COVID-19)
NCT04567615	**Nivolumab**<br>**Nivolumab**<br>+ relatlimab (2 doses) (LAG3 inhibitor)	II, randomized	≥ 2. line	Advanced/metastatic<br>Child Pugh A	250	ORR	September 2023, not yet recruiting

Clinical trial.gov identifier	Treatment	Phase	Line of treatment	Patient characteristics	Number of patients	Primary end points	Estimated study completion
NCT03647163	**Pembrolizumab** + VSV-IFNβ-NIS (oncolytic vesicular stomatitis virus)	I<br>Including non-SCLC	Any	Advanced/metastatic<br>Progression on PD-1/PD-L1 therapy	23	RR (expansion arm)	November 2020, recruiting
NCT03259867	**Pembrolizumab** + transarterial tirazamine embolization	II Including colorectal cancer	2. line	Advanced/metastatic<br>BCLC stage C<br>Child-Pugh score 5-7<br>Prior sorafenib	20	RR	December 2021, recruiting
NCT03519997	**Pembrolizumab** + bivatuzumab (Anti-CD44 antibody)	II	1. line	Advanced or metastatic, not amenable to curative treatment or locoregional therapy<br>Child-Pugh A	34	ORR	April 2021, recruiting
NCT02940496	**Pembrolizumab** + Elbasvir/Grazoprevir	II	2. line	Advanced with no curative option<br>Child-Pugh A	30	DLT	December 2021, active, not recruiting
NCT03836352	**Pembrolizumab** + DPX-survivac + low dose cyclophosphamide	II	Refractory	Advanced/metastatic<br>Expression of survirin	232	Disease progression	December 2022, recruiting

**Clinical trial.gov identifier**	**Treatment**	**Phase**	**Line of treatmen t**	**Patient characteristics**	**Numbe r of patient s**	**Primary end points**	**Estimated study completio n**
		Including other tumor types					
NCT03605706	**Camrelizumab** + FOLFOX<br>FOLFOX<br>Sorafenib	III	1. line	Advanced/metastatic<br>Child-Pugh A or B7	448	OS	February 2021, recruiting
NCT02795429	**Spartalizumab** + INC280 (MET inhibitor)<br>**Spartalizumab**	I/II	2. line	Recurrent or metastatic<br>Child-Pugh A<br>Prior sorafenib	87	DLT, MTD<br>ORR	December 2019, active, not recruiting
NCT04337463	**Toripalimab + ATG-008**	Not applicable (+ solid tumor)	Refractor y	BCLC B and C<br>Child Pugh A or B7	38	MTD, RP2D, ORR	March 2021, recruiting
NCT04612504	**Atezolizumab** + SynOV1 (Recombinant Oncolytic Adenovirus)	I/IIa	Refractor y	Locally advanced or metastatic<br>AFP positive	45	DLT, MTD, RR	August 2023, not yet recruiting

Clinical trial.gov identifier	Treatment	Phase	Line of treatment	Patient characteristics	Number of patients	Primary end points	Estimated study completion
NCT03257761	**Durvalumab** + guadecitabine (hypomethylating drug)	I<br>Including pancreatic bile duct and gallbladder cancer	2. line	Not amenable to potentially curative resection, local treatment<br>Child-Pugh score ≤ 7<br>Prior sorafenib	90	DLT, RR	February 2021, recruiting
NCT03099109	**LY3321367 (anti-TIM-3) +/- lodapolimab**	I	Refractory	Solid tumor	275	DLT	December 2019, active not recruiting
**PD-1/PD-L1 in combination with other treatment strategies**							
NCT04611165	**Nivolumab** + external beam radiation	II	1.or 2. line	Presence of major vascular invasion on dynamic CT or dynamic MRI<br>Child Pugh A	50	PFS	June 2022, recruiting
NCT04652440	**Tislelizumab** + RFA	I/II	1. line	Indication for RFA<br>BCLC B and C<br>Child Pugh A	30	Safety	December 2021, recruiting

**Clinical trial.gov identifier**	**Treatment**	**Phase**	**Line of treatment**	**Patient characteristics**	**Number of patients**	**Primary end points**	**Estimated study completion**
NCT04483284	**Camrelizumab** + TACE	II	Any	Unable or unwilling to undergo surgical treatment<br>BCLC B and C<br>Child Pugh A- B7	60	PFS	December 2020, recruiting?
NCT04724226	Cryoablation → **Camrelizumab** + apatinib	II	≤ 2. line	Advanced/metastatic<br>BCLC C<br>Child Pugh A	34	ORR	December 2021, not yet recruiting
NCT04592029	**Sintilimab** + bevacizumab + TACE	I	1.line	BCLC B and C<br>Child Pugh A-B7	36	AE, PFS	June 2022, recruiting
NCT04599790	**Sintilimab** + lenvatinib + TACE	II	1.line	Advanced<br>BCLC C<br>Child Pugh A-B7	30	OS	September 2022, recruiting
NCT03857815	SBRT + **Sintilimab**	II	1. line	Inoperable or unresectable	30	PFS	September 2020, recruiting

**Clinical trial.gov identifier**	**Treatment**	**Phase**	**Line of treatment**	**Patient characteristics**	**Number of patients**	**Primary end points**	**Estimated study completion**
NCT04547452	**Sintilimab** + stereotatic body radiation (SBRT)	II	2. line	Ineligible for curative intent therapy with surgical resection or liver<br>transplantation<br>Child Pugh A	84	PFS 24 weeks	July 2022, recruiting
NCT03864211	**Toripalimab**<br>Thermal ablation → **Toripalimab**	I/II (randomized)	Any	Ineligible for curative therapy<br>One lesion suitable for ablation<br>Prior PD-1/PD-L1 inhibitor<br>Extrahepatic disease allowed<br>Child-Pugh A or B7	120	AE<br>ORR	March 2021, recruiting
NCT04709380	Radiotherapy + **toripalimab**<br>vs<br>Sorafenib	III	Any	Advanced<br>BCLC C<br>Portal vein tumor thrombosis	85	TTP	January 2022, not yet recruiting

Clinical trial.gov identifier	Treatment	Phase	Line of treatment	Patient characteristics	Number of patients	Primary end points	Estimated study completion
				Child Pugh A-B7			
NCT04430452	Hypofractioned radiotherapy → **durvalumab + tremelimumab**	II	Any,	Advanced or metastatic; clinical indication for radiotherapy to any site<br>Child Pugh A - B7	30	ORR	November 2022, not yet recruiting

AE, adverse event; AEsi, adverse event of special interest; DLT, dose-limiting toxicity; BCLC, Barcelona Clinic liver cancer stage; MTD, maximum tolerated dose; RFS, recurrence-free survival; RR, response rate; ORR, overall response rate; OS, overall survival; PFS, progression-free survival; RFA, radiofrequency ablation; RP2D, recommended phase II dose; SAE, serious adverse event; TEAE, treatment emergent adverse event; TTP, time to progression; vs, versus.

Supplementary Table S2.

Ongoing studies with checkpoint inhibitors in locally advanced hepatocellular carcinoma

**Clinical trial.gov identifier**	**Treatment**	**Phase**	**Line of treatment**	**Patient characteristics**	**Number of patients**	**Primary end points**	**Estimated study completion**
**PD-1/PD-L1 monotherapy**							
NCT03916627	**Cemiplimab**	II (includes other cancer cohorts)	Any	Determined to be candidate for resection	94	Significant tumor necrosis	June 2020, recruiting
**PD-1/PD-L1 in combination with local treatment**							
NCT02837029	**Nivolumab** + Yttrium Y90-glass microspheres	I/Ib	Any	Advanced, not amenable to transplant or resection<br>Child-Pugh ≤ B8	35	MTD	July 2019, active, not recruiting
NCT03143270	**Nivolumab +** debTACE	I	1. line	Locally advanced, not eligible for surgical resection or liver transplant<br>Child-Pugh A	14	AE	April 2022, recruiting
NCT03099564	**Pembrolizumab** + Y90-radioembolization	I	1. line	Locally advanced, not eligible for surgical resection or liver transplant<br>Child-Pugh ≤ B7	30	PFS	July 2021, recruiting

Clinical trial.gov identifier	Treatment	Phase	Line of treatment	Patient characteristics	Number of patients	Primary end points	Estimated study completion
				Limited extrahepatic disease allowed<br>No prior TAE or TACE			
NCT03380130 (NASIR-HCC)	Yttrium Y90-glass microspheres → **nivolumab**	II	Any	Inoperable, candidate for local therapy<br>Child-Pugh A<br>No extrahepatic disease	40	AE	Completed
NCT03572582 (IMMUTACE)	**Nivolumab** + TACE	II	1. line	Not eligible for resection or local ablation<br>Child-Pugh A	49	ORR	December 2021, recruiting
NCT04340193	**Nivolumab + ipilimumab** + TACE<br>vs<br>**Nivolumab** + placebo +TACE	III	Not specified	Eligible for TACE	765	PFS, OS	February 2026, recruiting

Clinical trial.gov identifier	Treatment	Phase	Line of treatment	Patient characteristics	Number of patients	Primary end points	Estimated study completion
	vs<br>Placebo + TACE						
NCT03397654 (PETAL1)	TACE → **pembrolizumab**	I/II	1. line	Ineligible surgical resection or liver transplantation<br>Child-Pugh score < 7<br>No extrahepatic metastasis	26	TEAE	December 2021, recruiting
NCT03753659 (IMMULAB)	**Pembrolizumab** + RFA/MWA	II	1. line	Candidate for local ablation<br>Child-Pugh score ≤ 6	30	ORR	March 2022, recruiting
NCT03316872	**Pembrolizumab** + SBRT	II	2. line	Intrahepatic HCC amenable to SBRT<br>Child-Pugh A<br>Prior sorafenib	30	ORR	November 2022, recruiting
NCT04652492	**Tislelizumab** + TACE	II	1. line	BCLC C<br>Suitable for TACE<br>Child Pugh A- B7	72	TTP	November 2022, recruiting

Clinical trial.gov identifier	Treatment	Phase	Line of treatment	Patient characteristics	Number of patients	Primary end points	Estimated study completion
NCT04663035	**Tislelizumab** + ablation<br><br>vs<br><br>Ablation	II, randomized	Any	Recurrent lesions within 3 to 12 months	120	RFS, 1 year	December 2023, recruiting
NCT04517227	TACE or ablation → **durvalumab**	Pilot	Any	Not amenable for surgery or transplantation<br><br>Suitable for ablation<br><br>BCLC B<br><br>Child Pugh A-B7	30	AE, SAE, AEsi, discontinuation rate	August 2024, not yet recruiting
**PD-1/PD-L1 in combination with angiogenic drugs and local therapy**							
NCT04559607	TACE + **camrelizumab** + apatinib<br><br>vs<br><br>TACE	Not applicable, randomized	1. line	China liver cancer stage IIb-IIIa<br><br>Child Pugh A	188	PFS	September 2021, recruiting
NCT04479527	**Camrelizumab** + apatinib (VEGFR2 inhibitor) + TACE + FOLFOX	II	Any	Advanced<br><br>Surgical resection or local ablation is impossible	34	PFS	August 2023, not yet recruiting

**Clinical trial.gov identifier**	**Treatment**	**Phase**	**Line of treatment**	**Patient characteristics**	**Number of patients**	**Primary end points**	**Estimated study completion**
				Child Pugh A- B7			
NCT04599777	**Tislelizumab** + sorafenib + TACE	II	1. line	Advanced<br>Not amenable to curative therapies Amenable to TACE<br>Child Pugh A- B7	30	OS	September 2022, recruiting
NCT04541173	Y-90 TARE + **atezolizumab** + bevacizumab<br>vs<br>Y-90 TARE	II, randomized	1. line	Not amenable to transplantation or resection At least BCLC B<br>No extrahepatic disease	128	PFS	November 2021, recruiting
NCT04727307	**Atezolizumab** → RFA → **atezolizumab** + bevacizumab<br>vs<br>RFA	II, randomized	1. line	Eligible for ablation<br>No extrahepatic disease<br>Child Pugh A	202	RFS	January 2025, recruiting
NCT04712643	**Atezolizumab** + bevacizumab + TACE (on demand)	III	1. line	Eligible for TACE<br>No extrahepatic disease	342	"TACE PFS", OS	February 2025, recruiting

**Clinical trial.gov identifier**	**Treatment**	**Phase**	**Line of treatment**	**Patient characteristics**	**Number of patients**	**Primary end points**	**Estimated study completion**
	vs<br><br>TACE (on demand)			Child Pugh A			
NCT03778957 (EMERALD-1)	TACE + **Durvalumab** + placebo vs<br><br>TACE + **durvalumab** + Bevacizumab vs<br><br>**TACE** + placebo	III	Any	Not amenable for resection, transplantation, curative ablation<br><br>Amenable for TACE<br><br>No extrahepatic disease<br><br>Child-Pugh A + B7	600	PFS	September 2022, recruiting
**PD-1/PD-L1 in combination with CTLA-4 inhibitor and local therapy**							
NCT03203304	**Nivolumab** + **ipilimumab** + SBRT<br><br>**Nivolumab** + SBRT	I	Any	Ineligible for curative intent therapy with surgical resection or liver transplantation<br><br>Child-Pugh A<br><br>Extrahepatic disease allowed	50	ORR	August 2020, recruiting

**Clinical trial.gov identifier**	**Treatment**	**Phase**	**Line of treatment**	**Patient characteristics**	**Number of patients**	**Primary end points**	**Estimated study completion**
NCT04472767	**Nivolumab + ipilimumab +** cabozantinib + TACE	II	1. line	Not candidate for resection or transplantation<br>No extrahepatic disease<br>Child Pugh A orB7<br>PS 0-2	35	PFS at 6 months	March 2022, recruiting
NCT04605731	Radioembolization → **durvalumab + tremelimumab**	Ib	Any	Unresectable, locally advanced<br>No extra-hepatic disease<br>Child Pugh A	32	AE, DLT, ORR	October 2024, not yet recruiting
NCT03638141	**Durvalumab + tremelimumab** → TACE<br>**Tremelimumab** → TACE	II (parallel assignment)	1. line	"Have disease that responds to TACE"	30	ORR	November 2023, recruiting
NCT04522544 (IMMUWIN)	**Durvalumab + tremelimumab** + Y-90 SIRT<br>**Durvalumab + tremelimumab** + TACE	II, randomized	1. line	Not eligible for resection or local ablation<br>Child Pugh A	84	ORR	March 2024, recruiting

AE, adverse event; BCLC, Barcelona Clinic liver cancer stage; deb-TACE, drug eluting bead transarterial chemoembolization; DLT, dose-limiting toxicity; MWA, microwave ablation; NA, not applicable; ORR, overall response rate; OS, overall survival; PFS, progression-free survival; RFA, radiofrequency ablation; RFS recurrence-free survival; RR, response rate; SBRT, stereotactic body radiation; SIRT, selective internal radiation therapy; TACE, transarterial chemoembolization, TAE, transarterial embolization; TARE, transarterial radioembolization; TEAE, treatment emergent adverse event; vs, versus.

Supplementary Table S3.

Ongoing studies with checkpoint inhibitors in hepatocellular carcinoma in the preoperative or adjuvant setting

Clinical trial.gov identifier	Treatment	Phase	Line of treatment	Patient characteristics	Number of patients	Primary end points	Estimated study completion
**Preoperative setting, eligible for surgical resection**							
NCT03812562	Yttrium Y90-glass microspheres → **nivolumab** → resection	I		Eligible for resection<br>Child-Pugh ≤ B8	12	Recurrence rate	June 2021, recruiting
NCT03299946	**Nivolumab** + cabozantinib → resection	I		Locally advanced, borderline resectable<br>Adequate liver remnant and function	15	AE	December 2021, recruiting
NCT04658147	**Nivolumab**<br>**Nivolumab**<br>+ relatlimab (LAG3 inhibitor)	I, randomized	Preoperatively	Technically resectable<br>No extrahepatic spread, no nodal disease, and no bilateral left	20	No patients completing treatment and proceed to surgery	April 2025, not yet recruiting

Clinical trial.gov identifier	Treatment	Phase	Line of treatment	Patient characteristics	Number of patients	Primary end points	Estimated study completion
				and right branch portal vein involvement			
NCT03682276 (PRIME-HCC)	**Nivolumab + ipilimumab** → resection	I/II		Planned liver resection<br><br>No extrahepatic disease<br><br>Child-Pugh A	32	Delay of surgery, TEAE	December 2020, recruiting
NCT03630640	**Nivolumab** → electroporation → **nivolumab**	II	-	Eligible for electroporation<br><br>Child-Pugh A	50	Local recurrence free survival (2 years)	September 2020, recruiting
NCT03510871	**Nivolumab**<br><br>**Nivolumab + ipilimumab**	II, randomized	Prior treatment allowed	Potential for resection<br><br>Child-Pugh A	45	AE	September 2022, recruiting
NCT03222076	**Nivolumab**<br><br>**Nivolumab + ipilimumab**	II, randomized	Potential for resection<br><br>Child-Pugh A	Potential for resection<br><br>Child-Pugh A	30	AE	September 2022, active, not recruiting
NCT04425226 (PLENTY202001)	**Pembrolizumab +**<br><br>Lenvatinib<br><br>vs	Not applicable	Neoadjuvant<br><br>No prior systemic therapy	Exceeding Milan criteria before liver transplant	192	RFS	December 2022, recruiting

**Clinical trial.gov identifier**	**Treatment**	**Phase**	**Line of treatment**	**Patient characteristics**	**Number of patients**	**Primary end points**	**Estimated study completion**
	No intervention			Child Pugh A-B7			
NCT04701060	**Camrelizumab** + apatinib (VEGFR2 inhibitor)	II	Perioperative	Indication for resection<br><br>Child Pugh A	30	Safety, rate of enrollment (feasibility)	January 2022, not yet recruiting
NCT 03869034	**Sintilimab** + FOLFOX (transarterial infusion)	II		Locally advanced, potentially resectable<br><br>KPS ≥90<br><br>Child-Pugh A	40	PFS	March 2020, active, not recruiting
NCT04721132	**Atezolizumab** + bevacizumab → surgery	II	Preoperative	Resectable disease with no evidence of extrahepatic spread<br><br>Child Pugh A	30	Safety<br><br>Pathological response rate	December 2022, not yet recruiting
**Adjuvant setting**							
NCT03383458 (CheckMate 9DX)	**Nivolumab**<br><br>**Placebo**	III		Curative hepatic resection or ablation<br><br>Child-Pugh score 5 or 6	530	RFS	April 2022, recruiting

**Clinical trial.gov identifier**	**Treatment**	**Phase**	**Line of treatment**	**Patient characteristics**	**Number of patients**	**Primary end points**	**Estimated study completion**
NCT03337841 (AURORA)	**Pembrolizumab**	II	Neoadjuvant + adjuvant (until progression)	Radical cure possible by resection or RFA<br><br>Child-Pugh A	50	1-year RFS	October 2020, unknown
NCT03867084 (KEYNOTE-937)	**Pembrolizumab** vs<br><br>Placebo	III		Confirmed complete surgical resection or local ablation<br><br>Child-Pugh score 5-6	950	RFS<br><br>OS	July 2025, recruiting
NCT03722875	**Camrelizumab +** apatinib (VEGFR2 inhibitor)	Single arm, pilot		Complete surgical resection<br><br>BCLC stage B or C, no extrahepatic disease prior to resection<br><br>Child-Pugh A	45	RFS<br><br>OS	March 2019, unknown

**Clinical trial.gov identifier**	**Treatment**	**Phase**	**Line of treatment**	**Patient characteristics**	**Number of patients**	**Primary end points**	**Estimated study completion**
NCT03839550	**Camrelizumab + apatinib (VEGFR2 inhibitor) vs** Epirubicn + oxaliplatin intrahepatic	II, randomized		Complete response after surgical resection Child-Pugh A	200	RFS	February 2020?, not yet recruiting
NCT03914352	**Camrelizumab + portal vein thrombus** PD-l antibody + portal vein thrombus	Parallel assignment, randomized		No intra- or extrahepatic recurrence Child-Pugh A or B	40	OS DFS	December 2019?, recruiting
NCT04639180	**Camrelizumab** + apatinib vs Active surveillance	III	No prior treatment	Undergone a curative resection or ablation No extrahepatic spread Child Pugh A	674	RFS	July 2024, not yet recruiting

**Clinical trial.gov identifier**	**Treatment**	**Phase**	**Line of treatment**	**Patient characteristics**	**Number of patients**	**Primary end points**	**Estimated study completion**
NCT04653389	**Sintilimab** + TACE +/- radiotherapy → surgery → **sintilimab**	II	Neoadjuvant and adjuvant<br>No prior therapy	Technically resectable<br>Child Pugh A-B7	30	Event free survival	December 2022, recruiting
NCT04682210	**Sintilimab** + bevacizumab<br>vs<br>Active surveillance	III		Curative resection, high risk of recurrence	246	RFS	December 2023, not yet recruiting
NCT03859128	**Toripalimab**<br>vs<br>Placebo	II, III		No residual tumor<br>Child-Pugh A	402	RFS	October 2022, recruiting
NCT04649489	**Surgery → atezolizumab** + bevacizumab<br>**Atezolizumab** + bevacizumab	? randomized	No prior treatment	Feasibility for curative hepatic resection Portal vein tumor thrombosis<br>Child Pugh A	198	Time to treatment failure	September 2022, not yet recruiting

Clinical trial.gov identifier	Treatment	Phase	Line of treatment	Patient characteristics	Number of patients	Primary end points	Estimated study completion
NCT04102098 (IMBrave 050)	**Atezolizumab + bevazicumab**<br>vs<br>Active surveillance	III		No residual tumor after surgical resection or ablation<br>Child-Pugh A<br>High risk of recurrence	662	RFS	March 2023, recruiting
NCT03847428 (EMERALD-2)	**Durvalumab +** bevacizumab vs<br>**Durvalumab +**<br>Placebo vs<br>Placebo + placebo	III		Completed curative therapy (resection or ablation)<br>Child-Pugh score 5 or 6	888	RFS	June 2022, recruiting

AE, adverse event; DFS, disease-free survival; OS, overall survival; RFS, recurrence-free survival; TEAE, treatment emergent adverse event; vs, versus.
